# Single-cell RNA sequencing reveals tumor microenvironment characteristics in ovarian malignant Brenner tumor

**DOI:** 10.1016/j.gendis.2025.101635

**Published:** 2025-04-10

**Authors:** Yao Ge, Danni Huang, Yuliang Wu, Wei Huang, Runping Xu, Liqun Gao, Jing Guo, Zhongping Cheng

**Affiliations:** a Department of Gynecology and Obstetrics, Tenth People's Hospital, Tongji University School of Medicine, Shanghai 200040, China; b Department of Obstetrics and Gynecology, Ruijin Hospital Affiliated to Shanghai Jiao Tong University School of Medicine, Shanghai 200020, China

Brenner tumors are a relatively rare type of epithelial ovarian tumor, and while most Brenner tumors are benign, malignant Brenner tumors (MBT) account for about 1% of cases,^1^ and their tumor microenvironment remains largely unexplored. In this study, a 68-year-old woman was diagnosed with MBT based on the following pathologic findings: molecular pathology was positive for P67 and cytokeratin 7 (CK7) and negative for cytokeratin 20 (CK20), uroplakin, and P16. Single-cell sequencing was used to analyze cell heterogeneity in this rare case of early MBT and in two cases of early high-grade serous ovarian cancer (HGSOC). Six different cell types in the MBT tumor microenvironment were identified. Compared with HGSOC, the proportion of MBT immune cells was significantly reduced. Increased proportions of the Macrophages_1 subgroup and Fibroblasts_RGS5 subgroup further led to immunosuppression. The interaction of MBT cells with tumor infiltrating immune cells through co-stimulatory signaling, chemokine interactions, and immune checkpoint pathways enhanced the immunosuppressive tumor microenvironment.

A 68-year-old woman presented to the hospital three months following the detection of a pelvic tumor. A pelvic enhanced magnetic resonance imaging was performed, revealing a solid-cystic mass. Enhanced imaging showed clear enhancement of the solid component, cyst wall, and septa. Tumor markers, including CA125, CA199, CEA, CA153, NSE, and HE4, were elevated. Pathological examination showed a lobulated, encapsulated solid tumor on the right side, measuring 18 × 16 × 5 cm, with a mixture of cystic and solid areas. The tumor had a white, pouch-like or solid cut surface with visible red areas and contained purulent, viscous fluid. Nodular goiter-like structures were scattered along the capsule wall, with some papillary nodules, appearing white and soft upon sectioning. Immunohistochemical findings showed positivity for P67 and cytokeratin 7 (CK7) and negativity for cytokeratin 20 (CK20), uroplakin, and P16. The tissue was positive for P63, P53, cyclin D1, and EGFR. The Ki67 hotspot index in tumor cells was approximately 30%. Estrogen receptor (ER) and progesterone receptor (PR) were negative. The tissue also showed no expression of Gata-3, Pax-8, S-100, vimentin, or WT-1 proteins.

We conducted an in-depth single-cell analysis of ovarian cancer cells from this rare MBT case alongside two cases of HGSOC. Two cases of menopausal or postmenopausal women with pathologic stage I HGSOC were selected from the GSE184880 database as matched controls for MBT. In total, 28,777 cells were obtained from these samples, with 19,465 cells (68%) derived from MBT and 9312 cells (32%) from HGSOC. HGSOC samples were predominantly composed of T cells and natural killer (NK) cells (6094; 65.44%), whereas MBT samples contained a higher proportion of epithelial cells (17,966; 92.30%) and some macrophages (595; 3.06%), fibroblasts (375; 1.927%), and T/NK cells (328; 1.69%) (Fig. 1A, B).

**Figure 1:**
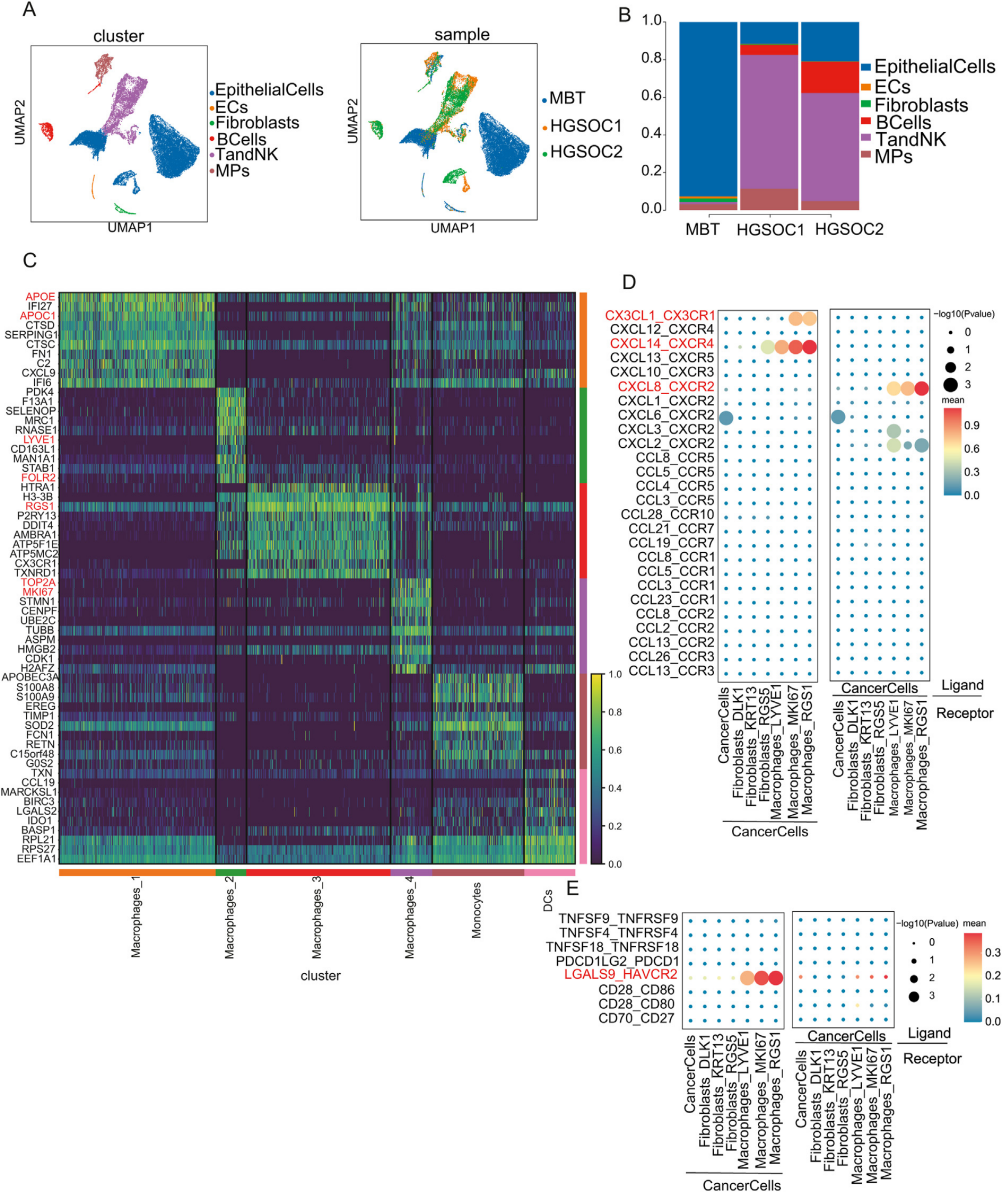
Single-cell transcriptomic analysis reveals different cell types in MBT and HGSOC tissue. **(A)** The UMAP shows the main cell types in MBT tissues and control HGSOC tissues. **(B)** Frequency distribution of cell types in different samples. **(C)** The heat map shows the scaled expression pattern of the top 10 marker genes in each mononuclear phagocyte system cell type, with color keys from blue to yellow indicating relatively low to high expression levels. **(D)** Comparison of important ligand–receptor pairs of chemokines between MBT tumor cells and other cells. **(E)** Comparison of important ligand–receptor pairs for immune checkpoint signaling between MBT tumor cells and other cells. MBT, malignant Brenner tumors; HGSOC, high-grade serous ovarian cancer; MPs, mononuclear phagocytes; ECs, endothelial cells; DCs, dendritic cells; NK, natural killer.

We further characterized the epithelial cells within the MBT tumor, which are all tumor cells, and the expression of marker genes in MBT cells was visualized by UMAP (Fig. S1A). Several genes were significantly up-regulated in MBT cells compared with HGSOC, including S100P, KIT13, and H3-3B (Fig. S1B). Notably, MBT cells were primarily enriched in pathways related to linoleic and linolenic acid metabolism, steroid hormone metabolism, and acetyl lipid metabolism (Fig. S1C). We further investigated that MBT cells exhibited lower enrichment in classical MHC-I and epithelial–mesenchymal transition pathways but higher enrichment in ferroptosis, hypoxia, and apoptosis pathways (Fig. S1C, D). In pseudotemporal analysis, MBT cells displayed an earlier developmental state compared with HGSOC tumor cells (Fig. S1E). These findings suggest that MBT cells have lower metastatic potential but are more susceptible to hypoxia, ferroptosis, and apoptosis.

The mononuclear phagocyte system cell population can be categorized into five distinct clusters, comprising one monocyte subtype, four macrophage subtypes, and one dendritic cell subtype (Fig. S2A). Macrophages_3 and Macrophages_2 formed the largest proportion in MBT (Fig. S2B). Macrophages_3 had a notable overexpression of regulator of G protein signaling 1 (RGS1), a known inhibitor of chemokine receptor signaling in lymphocytes. This subtype influences T-cell migration to tumors by dampening chemokine-mediated signaling, potentially leading to lymphocyte depletion.^2^ Macrophages_2, displaying an M2-like phenotype with markers CD163L1 and MRC1, expressed high levels of both FOLR2 and LYVE1 coding genes. FOLR2, which encodes the macrophage-specific folate receptor β, critical for folic acid transport, is an attractive therapeutic target for tumor-associated macrophages, highlighting Macrophages_2's immunosuppressive profile.^3^ Macrophages_1 was characterized by high expression of lipid metabolism genes APOE and APOC1, which were found to be enriched in HGSOC tissues (Fig. S2B). Macrophages_4 overexpressed cell cycle-related genes such as TOP2A and MKI67. Locus analysis confirmed that macrophages in MBT cells were in an early activation state (Fig. S2C). Functional enrichment analysis characterized that macrophages in MBT were mainly enriched in the cell adhesion pathway, linked to the heightened hypoxia in MBT tumor cells (Fig. S2D).

We further investigated the characteristics of interstitial fibroblasts, categorizing them into three subpopulations based on gene expression profiles: Fibroblasts_DLK, Fibroblasts_KRT13, and Fibroblasts_RGS5 (Fig. S3A). Notably, Fibroblasts_RGS5 represented the largest proportion in MBT cells (Fig. S3B). This RGS5^+^ fibroblast cluster, identified as tumor-associated fibroblasts,^4^ displayed a myofibroblast phenotype, marked by high ACTA expression (Fig. S3C, D). Gene scoring revealed that Fibroblasts_RGS5 clusters were more active in lipid metabolism (Fig. S3D).

Intensive communications across MBT cell clusters were characterized by the CellChat and CellPhoneDB. Subtypes such as Macrophages_RGS1, Macrophages_LYVE1, and Macrophages_MKI67 exhibited an enhanced IGF signaling pathway, contributing to tumor progression (Fig. S4A, B).^5^ Further analysis of chemokine communication revealed that CXCL14 and CX3CL1, secreted by MBT tumor cells, recruited macrophages through the CXCL14_CXCR4 and CX3CL1_CX3CR1 axes (Fig. 1D). Additionally, interactions between macrophages were also active. The interactions between T cells, NK cells, and Macrophages_RGS1 were notably active, with these immune cells closely linked through the CXCL14_CXCR4 and CXCL8_CXCR2 axes (Fig. S4C), suggesting potential chemotaxis between T cells, NK cells, and Macrophages_RGS1. In terms of inhibitory signals, we identified a prominent co-inhibitory signal via the LGALS9_HAVCR2 axis between macrophages and tumor cells (Fig. 1E). An LGALS9_HAVCR2 loop from Macrophages_RGS1 to other immune cells was also discovered (Fig. S4D). In summary, Macrophages_RGS1 may play a crucial role in creating an immunosuppressive microenvironment through co-stimulatory signaling.

In conclusion, our study provides key insights into the molecular and cellular mechanisms of the tumor immune microenvironment in patients with rare MBT. We have identified the presence of both naive and activated macrophages in MBT, which contribute to the immunosuppressive environment of MBT cells and the activation of signaling pathways linked to tumor progression.

## CRediT authorship contribution statement

**Yao Ge:** Writing – original draft, Methodology, Formal analysis. **Danni Huang:** Software. **Yuliang Wu:** Visualization. **Wei Huang:** Visualization. **Runping Xu:** Visualization. **Liqun Gao:** Visualization. **Jing Guo:** Writing – review & editing, Methodology. **Zhongping Cheng:** Writing – review & editing, Visualization, Validation, Methodology, Funding acquisition.

## Ethics declaration

The research involving human participants has been reviewed and approved by the Ethics Committee of Shanghai Tenth People's Hospital (ethics number: 24K198). The patients have provided written informed consent to participate in this study.

## Data availability

The datasets of HGSOC cellular heterogeneity analysis during the study are available from the Gene Expression Omnibus (GEO) repository (https://www.ncbi.nlm.nih.gov/geo/query/acc.cgi?acc=GSE184880).

## Funding

This work was supported by the 10.13039/501100001809National Natural Science Foundation of China (No. 82373269).

## Conflict of interests

The authors declared no conflict of interests.
