# Supplementary material for: Single-cell RNA sequencing reveals tumor microenvironment characteristics in ovarian malignant Brenner tumor
Source: Genes Dis. 2025 Apr 10;13(2):101635. doi: 10.1016/j.gendis.2025.101635 (PMC12664599; doi:10.1016/j.gendis.2025.101635)
Supplement: Multimedia component 1 [file mmc1.docx]

# Materials and methods

## **Clinical specimens collection**

We investigated a rare case of malignant ovarian brenner tumor using single-cell RNA sequencing, alongside cellular heterogeneity analysis incorporating data from the Gene Expression Omnibus (GEO) repository (https://www.ncbi.nlm.nih.gov/geo/query/acc.cgi?acc=GSE184880). Informed consent was obtained from the patient with malignant ovarian Brenner tumor at Shanghai Tenth People’s Hospital. A tumor specimen was surgically excised and subsequently confirmed as a malignant Brenner tumor by an expert pathologist.

## **Single‑cell RNA sequencing analysis**

Scanpy v1.8.1 was utilized for quality control, dimensionality reduction, and clustering within a Python 3.7 environment. After filtering, 28,777 cells were retained for downstream analyses, with an average expression of 2,105 genes and 7,285 UMIs per cell. The raw count matrix was normalized by total counts per cell and log-transformed into a normalized data matrix. The top 2,000 variable genes were selected using the "Seurat" flavor setting. Principal component analysis (PCA) was performed on the scaled variable gene matrix, and the top 20 principal components were used for clustering and dimensionality reduction. Cells were grouped into 21 clusters using the Louvain algorithm with a resolution parameter of 1.2. Uniform Manifold Approximation and Projection (UMAP) was applied for cluster visualization.

## **Differentially expressed genes (DEGs) analysis (scanpy)**

To identify differentially expressed genes (DEGs), we employed the scanpy.tl.rank_genes_groups() function, utilizing the Wilcoxon rank-sum test with default parameters. Genes expressed in more than 10% of the cells in at least one of the compared cell groups and exhibiting an average log (fold change) greater than 1 were designated as DEGs. The adjusted p-value was calculated, with a threshold of 0.05 set as the criterion for statistical significance.

## **Pathway enrichment analysis**

Gene Ontology (GO) and Kyoto Encyclopedia of Genes and Genomes (KEGG) analyses were conducted using the "clusterProfiler" R package (v3.16.1) to investigate the potential functions of MBT-related, immune-related, or fibroblast-associated genes. Pathways with an adjusted p-value below 0.05 were considered significantly enriched, and selected significant pathways were visualized as bar plots. For Gene Set Variation Analysis (GSVA) pathway enrichment, the average gene expression of each cell type served as input data. Gene Ontology gene sets encompassing molecular function (MF), biological process (BP), and cellular component (CC) categories were used as reference.

## **Cell-cell interaction analysis: CellPhoneDB and CellChat**

Cell-cell interaction (CCI) among MBT cells, macrophages, and fibroblasts was predicted based on known ligand-receptor pairs using CellphoneDB (v2.1.0) and CellChat (v0.0.2). The permutation number for establishing the null distribution of average ligand-receptor pair expression in randomized cell identities was set to 1,000. Expression of individual ligands or receptors was thresholded by a cutoff derived from the average log gene expression distribution across all genes in each cell type. Predicted interaction pairs with a p-value < 0.05 and average log expression > 0.1 were deemed significant and visualized using heatmaps and dot plots in CellphoneDB.

## **Cell Differentiation Potential Evaluation: CytoTRACE**

CytoTRACE v0.3.3 was utilized to predict the differentiation state of MBT cells and macrophages from single-cell RNA-sequencing data, based on gene counts and expression levels. This approach was applied to assess the differentiation potential of specific cell subpopulations.

## **Transcription factor regulatory network analysis**

The transcription factor network was constructed using pySCENIC (v0.11.0) with the scRNA expression matrix and transcription factors from AnimalTFDB. Initially, GRNBoost2 was employed to predict a regulatory network based on co-expression between regulators and targets. CisTarget was then applied to filter out indirect targets and identify transcription factor binding motifs. AUCell was subsequently used to quantify regulon activity within each cell. Cluster-specific transcription factor (TF) regulons were identified based on Regulon Specificity Scores (RSS), and the activity of these TF regulons was visualized through heat maps.
