# Supplementary material for: Single-cell RNA sequencing reveals tumor microenvironment characteristics in ovarian malignant Brenner tumor
Source: Genes Dis. 2025 Apr 10;13(2):101635. doi: 10.1016/j.gendis.2025.101635 (PMC12664599; doi:10.1016/j.gendis.2025.101635)

Figure S1. A unique class of tumor cells was revealed through the analysis of the composition of cell types in tumors in MBT.

(A) UMAP showed the expression levels of marker genes in MBT cell types.

(B) Volcanic map of gene expression changes in MBT and HGSOC tumor cells. The X-axis represents the natural logarithm of fold-changes (FC), and the Y-axis represents the negative logarithm of the adjusted P-value in base 10. The gray vertical and horizontal lines reflect the filtering conditions. Red and blue dots indicate significantly overexpressed genes and significantly underexpressed genes, respectively.

(C) Heat maps showing the top 20 up-regulated and down-regulated genes in MBT and HGSOC tumor cells. Colors from purple to yellow indicate relative expression levels from low to high.

(D) Violin plot showing Classical MHC-I, EMT, iron death, and ssGSEA scores for cancer cells from MBT and HGSOC samples. The P-values were calculated using a bilateral Wilcoxon rank sum test.

(E) Heat maps showing ssGSEA score levels of cancer cells in MBT and HGSOC samples.

(F) Using Monocle2 to predict the reconstructive trajectory of MBT and HGSOC ovarian cancer cells (color differentiation by subtype, characteristic gene expression, and pseudotime).


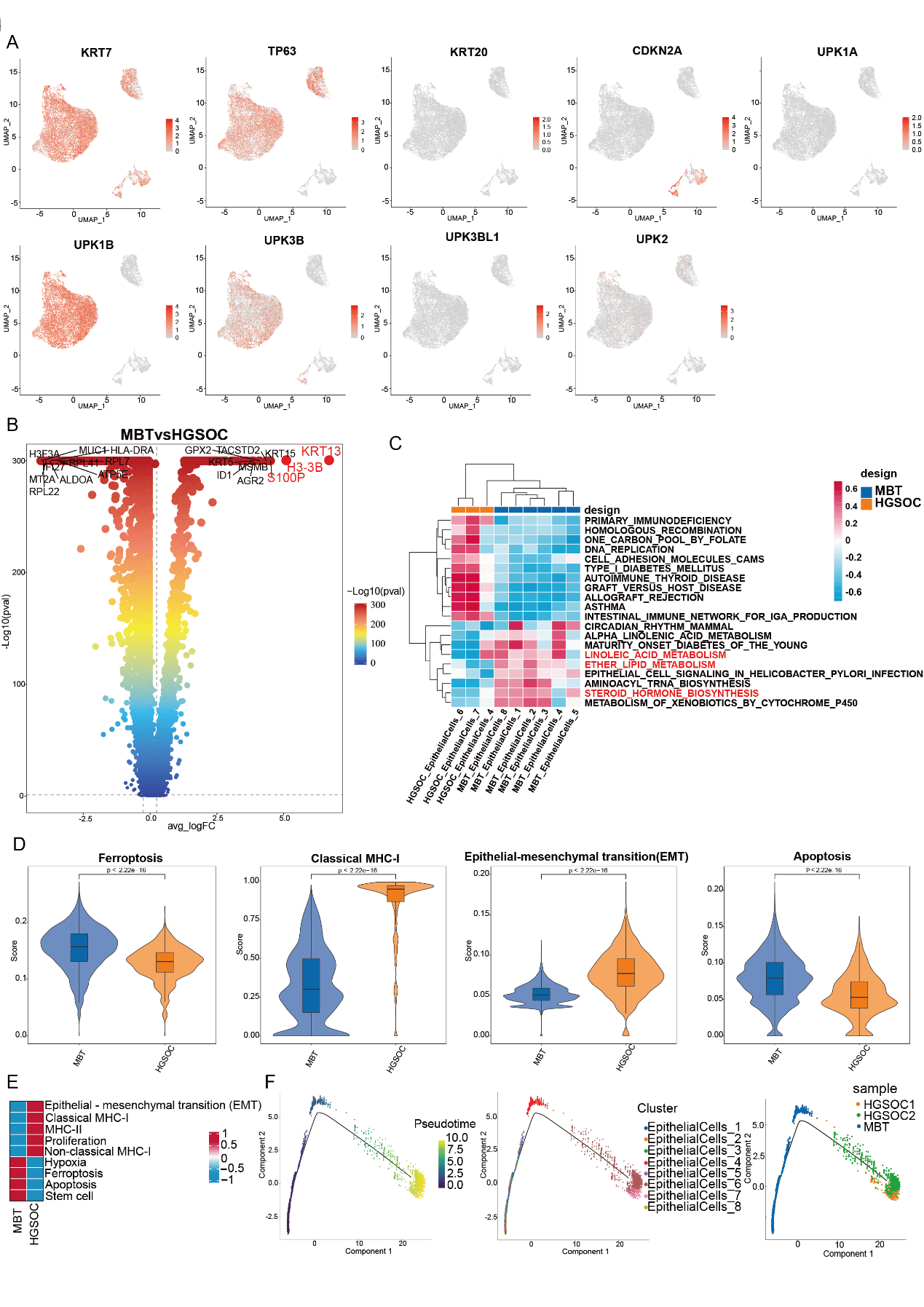

Supplement: Multimedia component 2 [file mmc2.docx]
