# Supplementary material for: Single-cell RNA sequencing reveals tumor microenvironment characteristics in ovarian malignant Brenner tumor
Source: Genes Dis. 2025 Apr 10;13(2):101635. doi: 10.1016/j.gendis.2025.101635 (PMC12664599; doi:10.1016/j.gendis.2025.101635)
Supplement: Multimedia component 3 [file mmc3.docx]

Figure S2. Characteristics of MPs cells in MBT and HGSOC tissues.

(A) UMAPs of all patient MPs cell, colored with identified cell subpopulations.

(B) Frequency distribution of different MPs cell types in MBT and HGSOC samples.

(C) Pseudotime analysis of different MPs cells in MBT samples.

(D) Functional enrichment analysis of macrophage -3 (Macrophages_RGS1) differential genes revealed the top variation items in the biological process of gene ontology. From blue to red, the P value decreases from large to small, and the area size of the circle represents the size of the quantity.


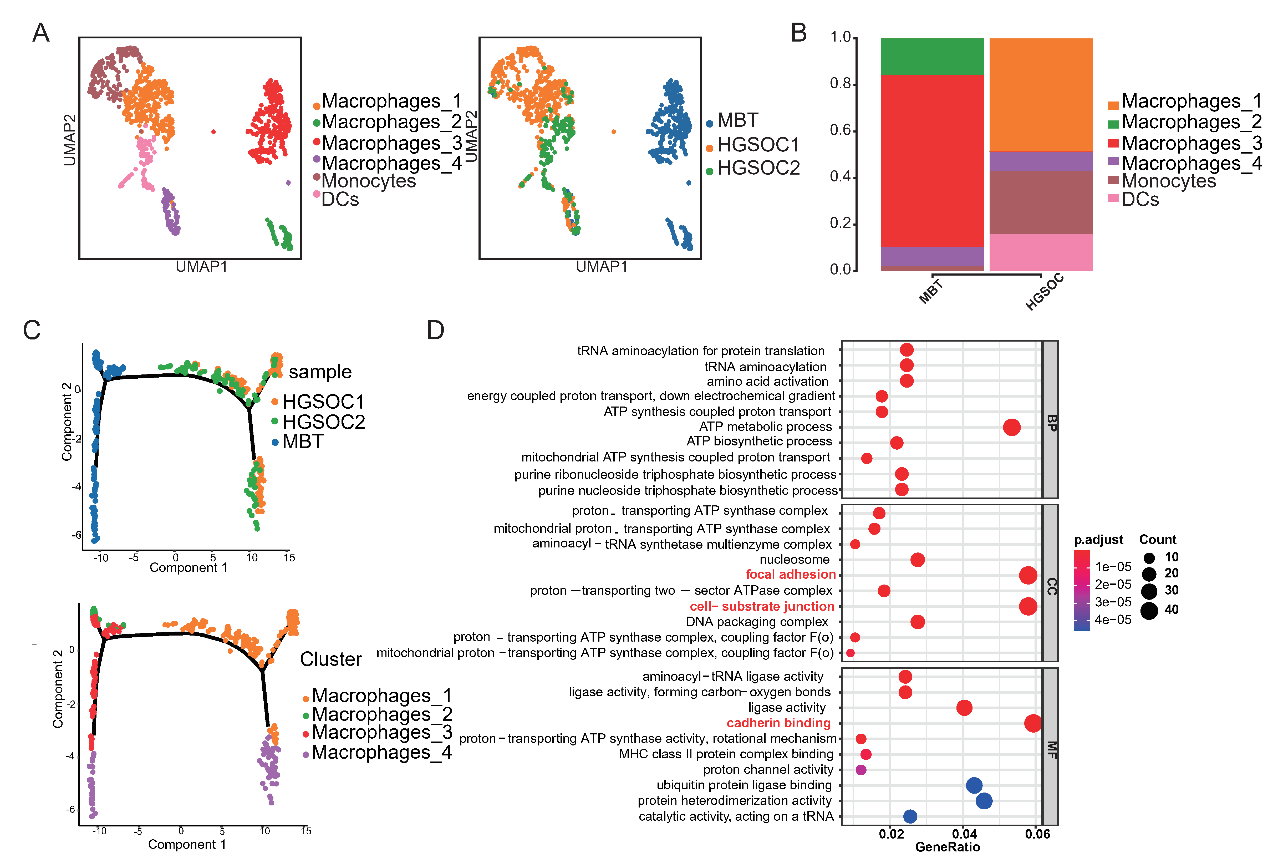
Celltypes: MPs cells, Mononuclear phagocyte system
