# Supplementary material for: Single-cell RNA sequencing reveals tumor microenvironment characteristics in ovarian malignant Brenner tumor
Source: Genes Dis. 2025 Apr 10;13(2):101635. doi: 10.1016/j.gendis.2025.101635 (PMC12664599; doi:10.1016/j.gendis.2025.101635)

FigureS3 Fibroblast clusters in MBT and HGSOC tumors.

(A) UMAP, color-divided clusters show 3 different clusters.

(B) Frequency distribution of fibroblast types in different samples.

(C) The heat map shows the scaled expression pattern of the top 10 marker genes in each fibroblast type. From blue to yellow, the level of gene expression increases gradually.

(D) The box diagram shows the ssGSEA scores of Myofibroblasts signature and Adipose derived signature from MBT and samples. The P-values were calculated using a bilateral Wilcoxon rank sum test.


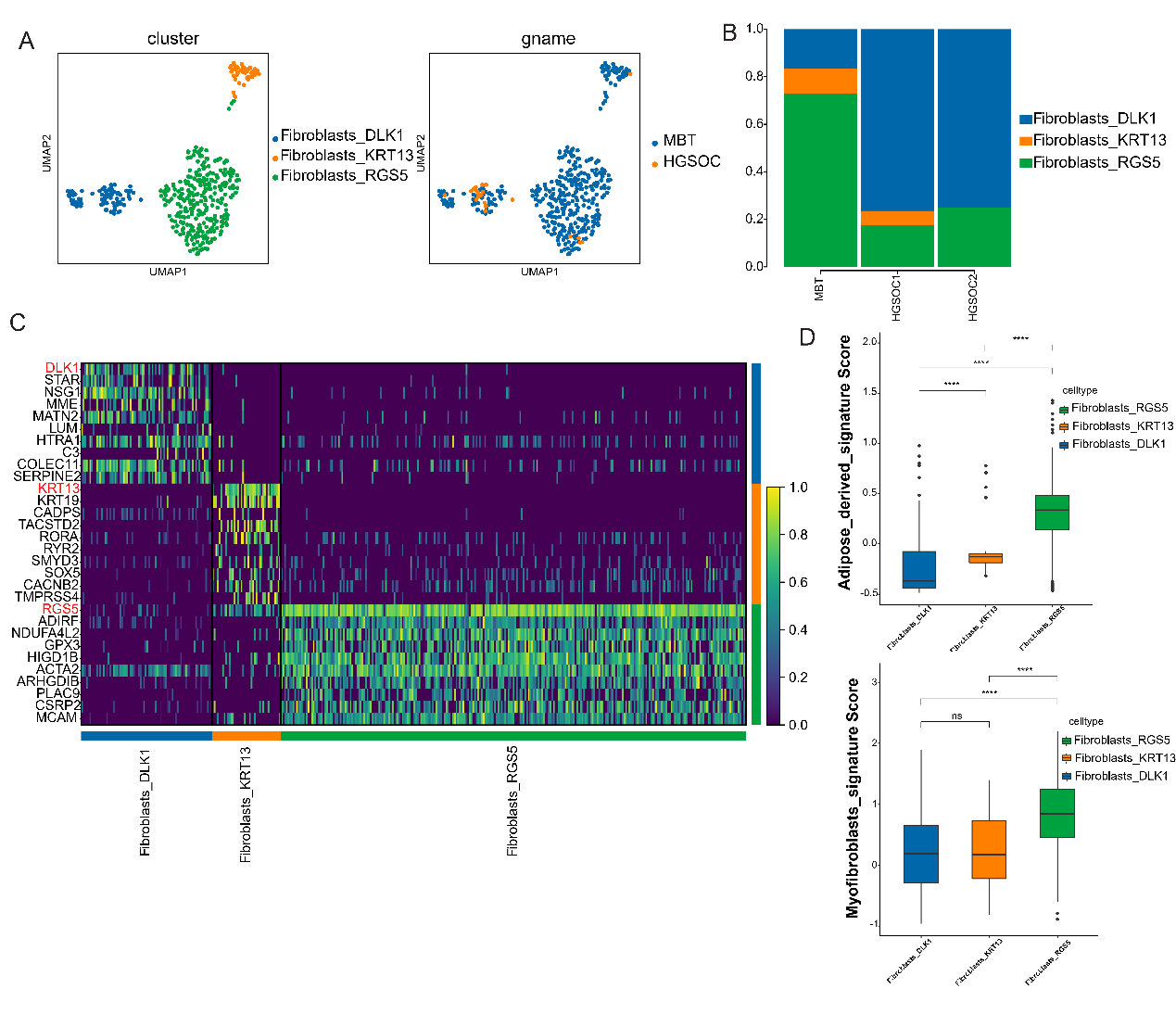

Supplement: Multimedia component 4 [file mmc4.docx]
