# Supplementary material for: Single-cell RNA sequencing reveals tumor microenvironment characteristics in ovarian malignant Brenner tumor
Source: Genes Dis. 2025 Apr 10;13(2):101635. doi: 10.1016/j.gendis.2025.101635 (PMC12664599; doi:10.1016/j.gendis.2025.101635)

Figure S4 MBT cells information exchange.

(A) The scatter plot shows a comparison of the output signal patterns of secretory cells.

(B) The scatter plot shows a comparison of incoming signal patterns.

(C) Comparison of important ligand-receptor pairs of chemokines between Macrophages_RGS1 and other cells.

(D) Comparison of important ligand-receptor pairs for immune checkpoint signalling between Macrophages_RGS1 and other cells.


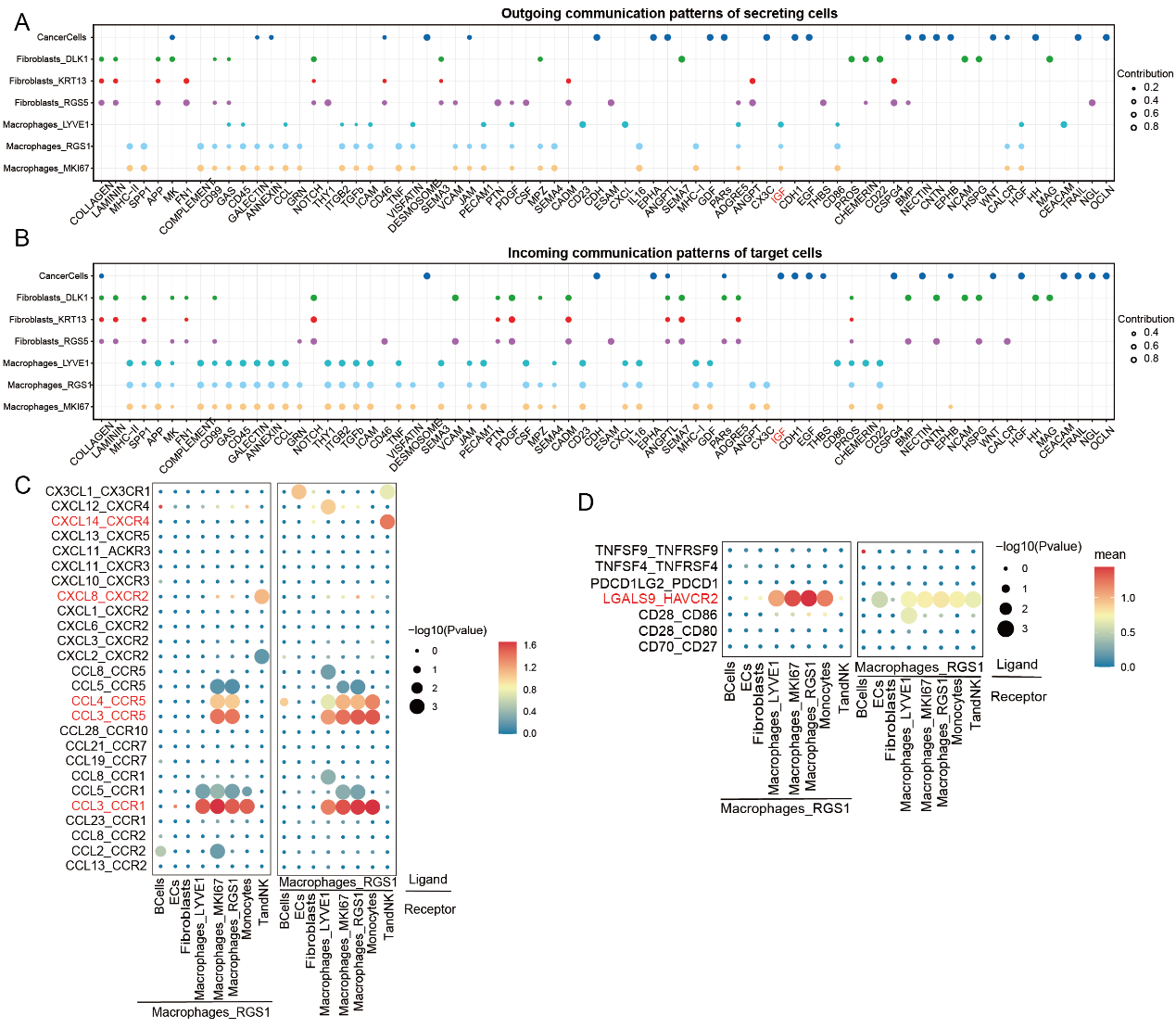

Supplement: Multimedia component 5 [file mmc5.docx]
